# Iron Acquisition and Metabolism as a Promising Target for Antimicrobials (Bottlenecks and Opportunities): Where Do We Stand?

**DOI:** 10.3390/ijms24076181

**Published:** 2023-03-24

**Authors:** Giovanni Stelitano, Mario Cocorullo, Matteo Mori, Stefania Villa, Fiorella Meneghetti, Laurent Roberto Chiarelli

**Affiliations:** 1 Department of Biology and Biotechnology “Lazzaro Spallanzani”, University of Pavia, Via A. Ferrata 9, 27100 Pavia, Italy; 2 Department of Pharmaceutical Sciences, University of Milan, Via L. Mangiagalli 25, 20133 Milano, Italy

**Keywords:** antimicrobial resistance, virulence factors, metallostasis, immunity, siderophores

## Abstract

The emergence of multidrug-resistant (MDR) and extensively drug-resistant (XDR) infections is one of the most crucial challenges currently faced by the scientific community. Developments in the fundamental understanding of their underlying mechanisms may open new perspectives in drug discovery. In this review, we conducted a systematic literature search in PubMed, Web of Science, and Scopus, to collect information on innovative strategies to hinder iron acquisition in bacteria. In detail, we discussed the most interesting targets from iron uptake and metabolism pathways, and examined the main chemical entities that exhibit anti-infective activities by interfering with their function. The mechanism of action of each drug candidate was also reviewed, together with its pharmacodynamic, pharmacokinetic, and toxicological properties. The comprehensive knowledge of such an impactful area of research will hopefully reflect in the discovery of newer antibiotics able to effectively tackle the antimicrobial resistance issue.

## 1. Introduction

The evolution and spread of antimicrobial resistance (AMR) have become major issues for global health and healthcare systems, causing almost 700,000 deaths/year. This number is expected to rise to 10 million by 2050, with an estimated cost for the global economy of 100 trillion dollars if no action is taken [1,2]. Furthermore, although several efforts have been made to increase global awareness and encourage best practices, AMR continues to rapidly emerge and spread, sometimes shortly after the introduction of a novel drug in clinical practice [3]. The AMR crisis has also been accelerated by the COVID-19 pandemic, mainly due to the increased use of antimicrobials in SARS-CoV-2 co-treatment. Moreover, the disruptions in healthcare systems and the slowdown of monitoring and treatment programs have contributed to worsening the situation [4].

There are several and different causes for the development of AMR, but the main determinant is most likely the misuse or overuse of antimicrobials, not only in healthcare, but also in agriculture and livestock. Bacteria have evolved different mechanisms that allow to develop drug resistance [5,6,7]. Moreover, many of the AMR mechanisms are probably “innate” to microorganisms and independent of contact with the antimicrobial agent [8]. This would explain the rapid emergence of resistance to new antibiotics, which would merely arise from pre-existing resistance factors within the microbial genome [9]. In this context, it is noteworthy that several genetic determinants for AMR are mobile and can be subjected to intra- and inter-species horizontal transfer [10]. This, together with the fact that antimicrobials usually target essential pathways, imposing a selective pressure that favors resistance, further promotes the spread of antibiotic resistance genes (ARGs) [11].

Another issue of AMR refers to the dissemination and transmission of ARGs from hotspots to the environment. These hotspots are not limited to medical settings, but include several other anthropogenic sources, such as wastewater systems, manufacturing plants, aquaculture, and breeding facilities [12], leading to the need of reliable surveillance and risk assessment procedures [13,14].

Therefore, several approaches are needed to fight AMR, including the development of novel antibiotics or vaccines, and the design of alternative strategies [15]. A promising option among these innovative solutions is the anti-virulence therapy, which is the use of compounds targeting pathways that are non-essential for microbial growth, but required for pathogenesis [16]. Most notably, because this approach aims to prevent the attack of a pathogen rather than kill it, anti-virulence compounds do not exert a selective pressure, thus preventing resistance phenomena [17].

Within this review, we will focus our attention on strategies aimed at targeting bacterial iron metabolism or iron uptake mechanisms, which represent promising targets for the development of innovative anti-virulence and antimicrobial agents.

## 2. Bacterial Mechanisms of Iron Uptake

Being an important cofactor for enzymes involved in fundamental cellular processes, iron is essential for bacteria; however, despite its relative abundance, it has a relatively low bioavailability. In detail, iron is usually present in its oxidized form (Fe^3+^), which is quite insoluble at neutral or slightly basic pHs. Moreover, in humans iron is prevalently bound to iron-storage or -transport proteins, such as ferritin, lactoferrin and transferrin, or complexed with hemoproteins or enzymes [18]. As a result, bacteria, and particularly pathogenic bacteria, have evolved several mechanisms for its acquisition from the environment [19]. Indeed, one of the first host defense strategies is the limitation of iron and other essential nutrients, an innate process known as nutritional immunity [20].

The most common iron acquisition strategies include the uptake of organic compounds binding iron, such as heme or citrate, and the production of siderophores or hemophores. Siderophores are secondary metabolites secreted into the external environment, where they bind Fe^3+^, forming soluble complexes that are then internalized through specific receptors [21]. Some bacteria can also scavenge heme iron from the host through the production of hemophores and/or specific transport systems for both heme-binding proteins and free heme [22].

The biosynthesis of siderophores occurs mainly through non-ribosomal peptide synthetases and/or polyketide synthase domains that work in concert, although there are few cases of molecules synthesized by other pathways [19]. To maintain cellular iron homeostasis, the synthesis of siderophores, as well as their release and uptake mechanisms, are tightly regulated. The most common control element is the ferric uptake regulator Fur. This transcriptional repressor forms a complex with cytosolic Fe^2+^ in non-limiting iron conditions, and then binds to the promoter of genes associated to siderophore biosynthesis or regulatory activators, repressing their transcription. In iron starvation, the repression by Fur is removed, thus allowing for the synthesis of siderophores [23].

**Figure 1:**
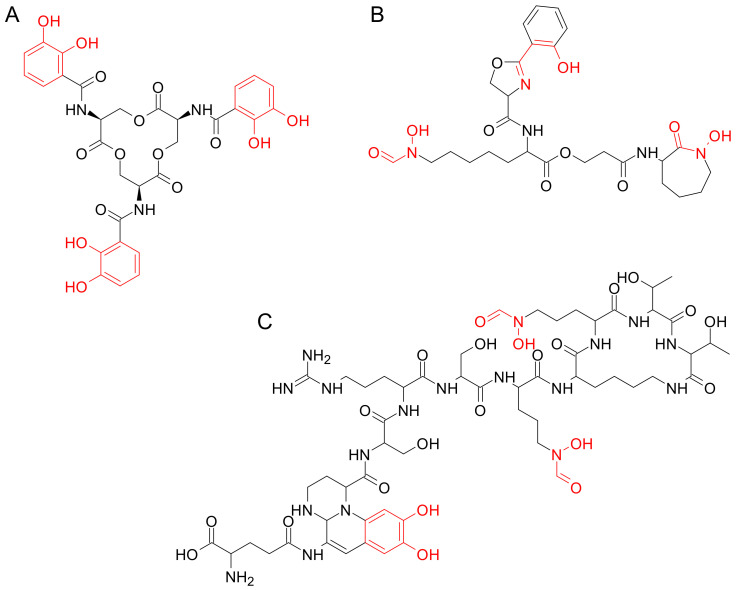
Chemical structures of representative siderophores: (**A**) catecholate enterobactin (PubChem CID:34231); (**B**) hydroxamate mycobactin (PubChem CID: 3083702); (**C**) mixed-type pyoverdine (PubChem CID: 57012495). The moieties involved in iron chelation are represented in red.

According to their chemical nature, siderophores can be classified into catecholates, hydroxamates, and mixed-type siderophores, which can have different chemical groups including, among others, carboxylates, quinones, oxazolines, and imidazoles (Figure 1) [24,25]. These molecules show a higher affinity for ferric (Fe^3+^) iron compared to ferrous (Fe^2+^) iron or other bivalent and trivalent metal ions. In detail, iron in siderophores is usually coordinated by oxygen atoms, in an octahedral geometry, which allows for the arrangement of six ligands around the Fe center. In some cases, the octahedral conformation is distorted, in others, the coordinating groups contain nitrogen or sulfur atoms; however, these derivatives generally show lower affinity for Fe^3+^ [24].

Siderophores are produced by both Gram-positive and Gram-negative bacteria, which possess different mechanisms for its uptake. In Gram-positive bacteria, the internalization of the siderophore–iron complexes is achieved by a membrane anchored protein and an ATP-binding cassette (ABC) transporter. The transport system in Gram-negative bacteria usually consists of an outer membrane receptor, the TonB system, which shuttles the siderophore–iron complex into the periplasmic space. Here, it binds to a specific periplasmic-binding protein and is moved to the cytoplasm through an inner membrane ABC transporter [24].

Mycobacteria are peculiar because they possess two siderophores, the relatively soluble carboxymycobactin and the insoluble mycobactin [25]. These molecules are exported across the inner membrane through MmpL4/MmpS4 and MmpL5/MmpS5 proteins. Then, mycobactins remain anchored to the outer membrane or into the cell wall, while carboxymycobactins are exported extracellularly to scavenge iron from the host iron-containing proteins. Carboxymycobactins can transfer iron to mycobactins, which transport it across the cell wall. Subsequently, thanks to periplasmic-binding proteins, the metal is shuttled through the periplasmic space to the inner membrane, and finally transferred into the cytoplasm by the IrtA/IrtB ABC-transporter [25].

Therefore, given the essentiality of iron for pathogenic bacteria, and the peculiarity of the mechanisms evolved for its uptake, which usually involves enzymes and proteins absent in humans, its metabolism has been considered for the development of novel antimicrobial and anti-virulence compounds.

## 3. Antimicrobial Strategies Involving Iron Metabolism

Different strategies, directly or indirectly, involving iron metabolism can be exploited to develop antimicrobial compounds. These approaches are based on the depletion of the environmental iron, the use of mimetic metals iron competitors, such as gallium, the inhibition of enzymes involved in siderophore biosynthesis and/or environmental iron-sensing mechanisms, and the exploitation of iron uptake systems for delivering drugs into the cell.

### 3.1. Depleting Environmental Iron

An effective strategy to inhibit bacterial growth could be the limitation of iron availability using chelators. The first evidence of the possibility of inhibiting microbial growth through this approach arose from the studies by Snow on mycobactins [26]. In detail, it was found that the mycobactins produced by a mycobacterium can antagonize the growth of other mycobacterial species. For instance, mycobactin S, produced by *M. smegmatis*, can inhibit *M. tuberculosis*, which instead produces mycobactin T [26]. Interestingly, the two siderophores differ only for one stereogenic center [27].

There are several iron chelators, such as deferoxamine, deferiprone, and deferasirox, which are currently used for the treatment of iron overload in patients with thalassemia. Therefore, these drugs have been investigated for a potential repurposing as antimicrobials, but without significant results [28]. Moreover, these chelators present toxicity issues, mainly due to their lack of selectivity for extracellular iron. As a result, they can also access the intracellular iron pool of the host, causing side effects [28]. Other small molecules able to chelate iron with high affinity were synthesized, but all of them showed only a low-to-moderate antimicrobial activity [29,30,31].

**Figure 2:**
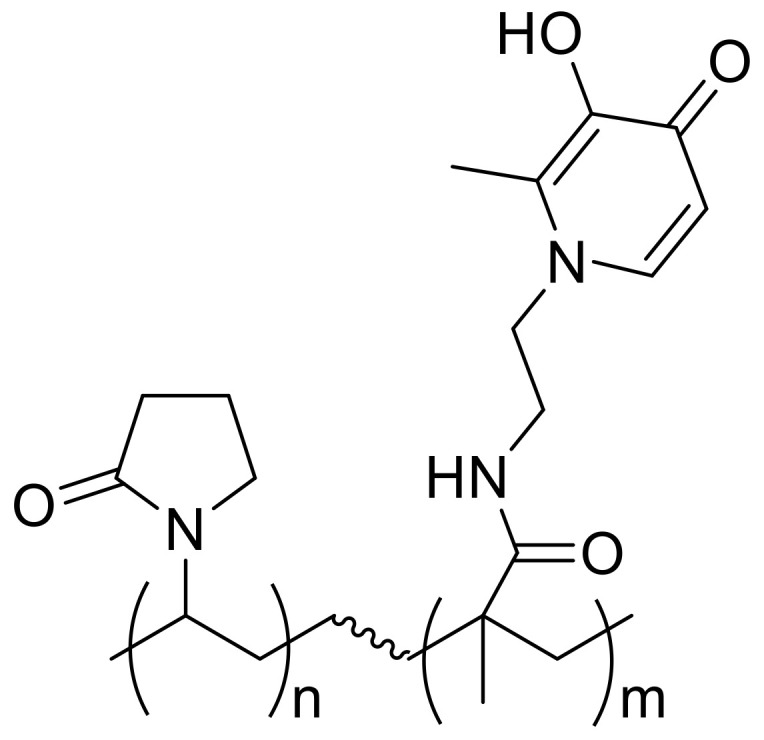
Chemical structure of the 3-hydroxypyridin-4-one of DIBI.

To overcome these issues, Ang and co-workers designed a 9 kDa 3-hydroxypyridin-4-one polymer, named DIBI (Figure 2) [32]. The idea was that a high-molecular-weight polymer could be conceivably less internalized in host cells, thus limiting the toxic effects. Moreover, the large polymeric chelator around Fe^3+^ ion would create a spatial barrier, reducing the accessibility of iron to bacterial siderophores.

DIBI was synthesized to selectively coordinate three Fe^3+^ ions. It was highly soluble in water and showed good antimicrobial activity against different Gram-positive bacteria, such as *Staphylococcus aureus* and methicillin-resistant *S. aureus* (MRSA) strains, [33,34,35] and against the Gram-negative *Acinetobacter baumanii* [36].

Following a similar approach, Abbina and co-workers synthesized a series of very-high-molecular-weight Fe^3+^ chelators, by conjugating the hexadentate chelator, *N,N-*bis(2-hydroxybenzyl)ethylenediamine-*N,N*-diacetic acid (HBED), with high-molecular-weight polyglycerol (HPG) [37]. Exploiting the characteristics of HPG (high water solubility, biocompatibility, and numerous functional end-moieties), they obtained different 100–200 kDa HPG-HBED conjugates, bearing a high number of chelating groups, ranging from 25 to 244. This characteristic led to an improved solubility of the macrochelator and to a significantly lower toxicity, with respect to the native HBED. Despite HBED-HPGs not showing any activity against *Pseudomonas aeruginosa*, a promising bacteriostatic activity was found against *Staphylococcus aureus* [37].

### 3.2. Gallium as an Iron Mimetic

Another interesting strategy to deprive bacteria of iron is the use of a metal that can act as an iron competitor. In this context, a good candidate is gallium. The physico-chemical properties of Ga^3+^ are very similar to those of Fe^3+^. As a result, it shows a good affinity for iron-dependent enzymes, but it cannot be reduced in physiological conditions. Therefore, being unable to participate in redox reactions, Ga^3+^ leads to the inhibition of several enzymes, interfering with essential bacterial pathways [38].

**Figure 3:**
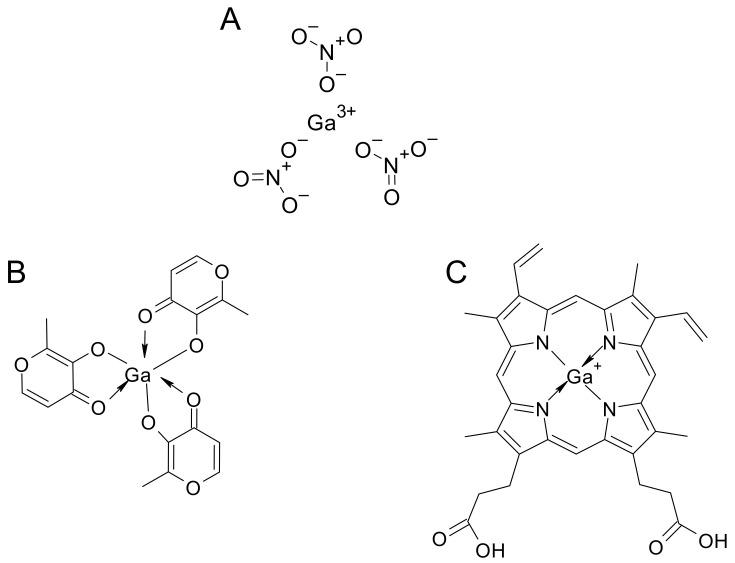
Chemical structures of gallium compounds belonging to the three different groups: (**A**) first-generation gallium nitrate; (**B**) second-generation gallium maltolate; (**C**) third-generation gallium protoporphyrin IX.

Gallium compounds can be divided into three groups: first, second, and third generation (Figure 3) [39]. Gallium nitrate (GaN, Figure 3A), the first Ga^3+^ compound that entered clinical trials, is a representative of the first generation. In second-generation compounds, Ga salts were attached to specific ligands, such as maltolate (GaM, Figure 3B), to increase their bioavailability. Finally, in third-generation compounds, gallium was complexed with different ligands, such as pyridine, hydrazones, thiosemicarbazones, and protoporphyrins [39].

In particular, Ga-(III)-protoporphyrin IX (GaPPIX, Figure 3C) has been deeply studied as a heme mimetic for the development of antimicrobial molecules. This complex was conceived to be internalized by the pathogen and interfere with the pathways involving heme. Interestingly, these derivatives proved to be particularly effective as antimicrobial compounds [40,41].

As previously mentioned, the repurposing of gallium compounds as antimicrobials exploits the fact that iron is required by most pathogenic bacteria. For instance, gallium can compete with iron for siderophores, thus inhibiting iron uptake and/or internalization. Moreover, gallium could interfere with the regulation of iron acquisition, repressing the transcriptional regulators for the biosynthesis of siderophores [42,43].

Several studies have recently demonstrated the activity of gallium compounds against different Gram-negative and Gram-positive bacteria, including ESKAPE species (*Enterococcus faecium*, *Staphylococcus aureus*, *Klebsiella pneumoniae*, *Acinetobacter baumannii*, *Pseudomonas aeruginosa*, and *Enterobacter* species), the most common nosocomial-infection-causing bacteria [41,44,45,46,47]. These molecules have also proved to be effective against opportunistic pathogens that cause lung infections in patients with cystic fibrosis or chronic obstructive pulmonary disease [41,42,44,45,46,47,48,49,50,51], including mycobacteria [40,52].

In detail, a pilot human study demonstrated the efficacy of intravenous gallium nitrate in patients with cystic fibrosis suffering from a chronic *P. aeruginosa* infection [49]. However, in an in vivo rat model, intravenously-administered GaN, showed poor pharmacokinetic properties and was associated with side effects, such as nephrotoxicity [53]. Hence, the possibility of intratracheal administration was suggested to directly deliver gallium into the lungs [53]. To this end, different methods to formulate inhaled gallium have been developed, such as the use of β-cyclodextrin/Ga(III) nanocarriers, obtained by encapsulating Ga-protoporphyrins into β-cyclodextrin [45,54], or the encapsulation Ga^3+^ in hyaluronic acid/chitosan nanoparticles [51], which were very promising in terms of bioavailability, efficacy, and tolerability.

The antimicrobial potential of gallium-containing compounds has also been exploited to develop hydrogels for the treatment of infected wounds [55,56,57]. Hydrogels are increasingly considered as attractive wound dressings materials for their ability to provide an adequate porosity for tissue repair, as well as a high biocompatibility and degradability. In this context, hydrogels can be useful for the topical delivery of antimicrobials, even at high dosages, directly to the infection site, increasing their efficacy [55]. A first example was the incorporation of Ga-protoporphyrins in a surgical hydrogel, together with the iron chelator, defeniprone [55]. This hydrogel showed a significant antimicrobial activity, as well as an antibiofilm effect against different bacteria, including *S. aureus* and MRSA clinical isolates, *Staphylococcus epidermidis*, *P. aeruginosa,* and *Acinetobacter johnsonii*. [55]. Different types of hydrogels and gallium compounds have been combined, with the aim to improve the efficacy of these antibacterial wound dressings. For instance, the combination of Ga(NO_3_)_3_ and silk fibroin hydrogel showed a good efficacy in an in vivo *P. aeruginosa* murine infection model [56], while another group demonstrated the in vivo efficacy of alginate-Ga^3+^ hydrogels against *E. coli* and *S. aureus* infections [57].

### 3.3. Inhibiting Siderophore Biosynthesis

Another efficient way to inhibit bacterial iron uptake is to block the production and export of siderophores by interfering directly with their biosynthesis or regulation systems.

This approach has been particularly applied for the development of antimycobacterial compounds, particularly against *M. tuberculosis*, targeting the peculiar biosynthetic pathway of mycobactins [58].

**Figure 4:**
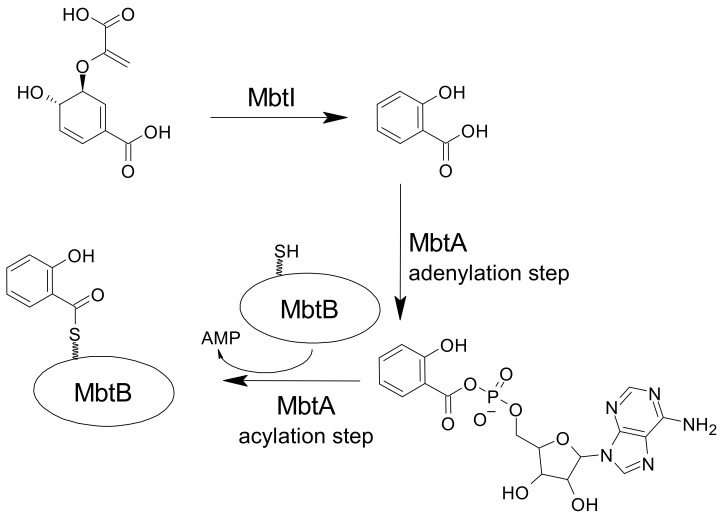
The first two reactions of the biosynthesis of mycobactins, namely the conversion of chorismate to salicylate (catalyzed by MbtI), and the activation of salicylate by adenylation and its subsequent transfer to MbtB (catalyzed by MbtA), are the most exploited for the development of inhibitors of this pathway.

The first enzyme involved in the biosynthesis of mycobactins is a salicylate synthase (MbtI in *M. tuberculosis*) that converts chorismic acid into salicylic acid, which then becomes the substrate of the mixed non-ribosomal peptide synthase-polyketide synthase MbtA-N. MbtA activates salicylic acid via an acyl adenylate intermediate, catalyzing its transfer to the N-terminal thiolation domain of MbtB (Figure 4). MbtB and MbtE attach the serine and lysine residues and then, together with MbtC and MbtD, the two malonyl CoA residues. Finally, the terminal lysine is incorporated by MbtF, and further modified by MbtK and MbtG, to give the final mycobactin scaffold [25,58]. The first two enzymes of this pathway, MbtI and MbtA, are the most investigated, with to the aim of developing specific inhibitors [25,58].

The majority of mycobactin biosynthesis inhibitors target MbtA. This bifunctional enzyme converts salicylate to Sal-AMP, which is subsequently loaded on the phosphopantetheinylation domain of MbtB [59].

**Figure 5:**
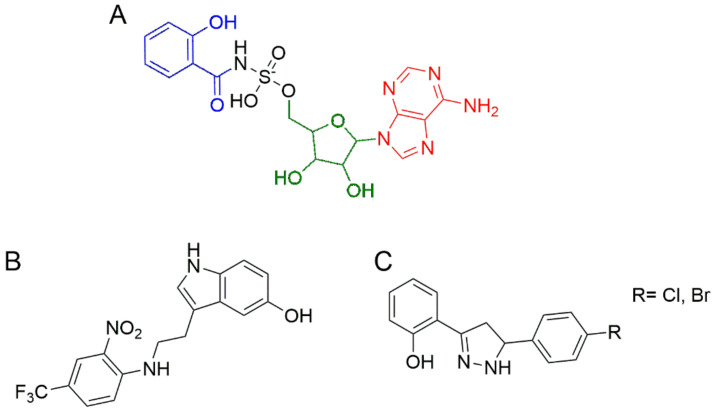
Chemical structures of MbtA inhibitors with antitubercular activity: (**A**) 5′-O-N-salicylsulfamoyl adenosine, with the salicylate moiety in blue, the sugar in green, and the nucleobase in red; (**B**) 5-hydroxy-indol-3-ethylamino-(2-nitro-4-trifluoromethyl)benzene obtained by whole-cell screening, combined with a target-based approach; (**C**) 3-(2-hydroxyphenyl)-5-(aryl)-pyrazolines derived from the mycobactin structure by rational design.

Most of the known MbtA inhibitors are designed to mimic the structure of Sal-AMP, with the aim to block the adenylation activity. The first reported analog was 5′-O-N-salicylsulfamoyl adenosine (Sal-AMS), in which a sulfonamide linkage replaced the phosphate ester group [60] (Figure 5A). Sal-AMS displayed a potent inhibition of MbtA and a promising activity against *M. tuberculosis* in iron-limiting conditions. Moreover, this compound showed good efficacy in a murine model, but its pharmacokinetic profile demonstrated a poor oral bioavailability [61]. Nevertheless, based on the good in vivo activity, several drug discovery campaigns were launched to improve the pharmacokinetic profile of Sal-AMS. In detail, the salicylate residue [62,63,64,65], the sugar moiety [52,66], and the purine group [67,68] were investigated through different analogs.

These studies led to several derivatives with improved features, both in terms of antitubercular activity and oral bioavailability. Among the attempted substitutions, the replacement of the aromatic hydroxyl group of the salicylate residue with a fluoro-substituted cinnolone moiety [65], the difluorination of the ribose group [66], and the incorporation of bulky phenyl rings at the adenine unit [68], led to the greatest improvement of the pharmacokinetic profile.

However, because most of Sal-AMS analogs showed poor drug-like properties, further studies were focused on the identification of non-nucleoside-based MbtA inhibitors. For this purpose, different approaches were adopted. For instance, by combining phenotypic screening and target-based drug discovery, Ferguson and co-workers identified 5-hydroxy-indol-3-ethylamino-(2-nitro-4-trifluoromethyl)benzene (Figure 5B) as a high-affinity ligand of MbtA, having a good antimycobacterial activity [69]. Another study, which exploited a rational design based on the mycobactin structure, led to the disclosure of novel 3-(2-hydroxyphenyl)-5-(aryl)-pyrazolines (Figure 5C), active against *M. tuberculosis* and non-tuberculous mycobacteria (NTM), and with very good pharmacokinetic properties [58].

**Figure 6:**
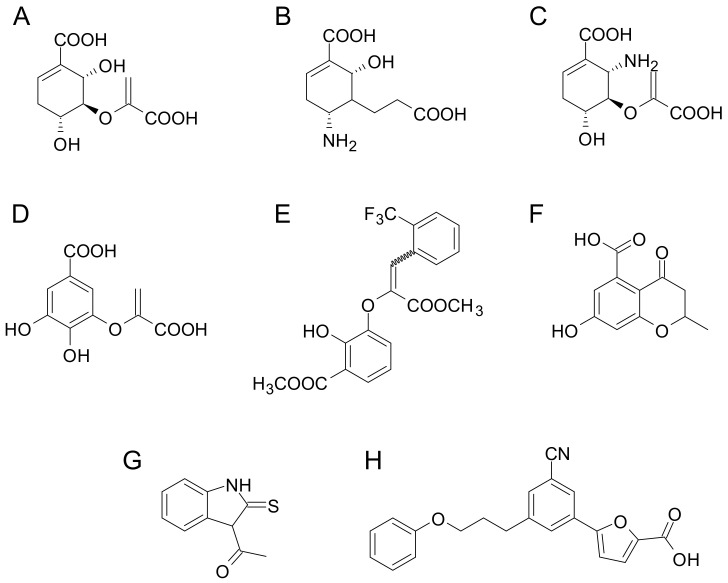
Chemical structures of the most active inhibitors of MbtI: transition-state analogs (**A**–**F**) and non-transition-state derivatives, namely benzimidazole-2-thione (**G**) and phenylfuran carboxylate (**H**) compounds.

The other most studied enzyme involved in mycobactin biosynthesis is the magnesium-dependent salicylate synthase MbtI, which catalyzes the conversion of chorismate to salicylate (Figure 4). The first inhibitor that was developed was the transition-state analog, 5-(2-carboxyallyl)-4,6-dihydroxycyclohex-1-ene-1-carboxylic acid (Figure 6A), which exhibited a promising activity against the isochorismate synthase [70]. On this basis, several efforts were dedicated to the synthesis of different transition-state analogs bearing the same scaffold (Figure 6B,C) [71,72], or the trihydroxybenzoate (gallate) (Figure 6D) [73,74,75], 3-phenylacrylate (Figure 6E) [74], or chromane (Figure 6F) [76] moieties. However, none of them displayed improved activity against MbtI.

Contextually, several non-transition-state inhibitors were disclosed by different approaches. For instance, using a high-throughput screening, based on the enzymatic activity of MbtI, Vasan and colleagues identified several benzisothiazolones, diaryl sulfones, and benzimidazole-2-thiones, with the latter being the most active class of compounds (Figure 6G) [77]. Similarly, an in silico virtual screening of a commercially available library led to the identification of a 5-phenylfuran-2-carboxylate compound, highly active against MbtI, and showing a moderate antitubercular activity in iron-depleted conditions, associated to a reduction in siderophore production [78]. Subsequent structure–activity relationship investigations were performed on this structure [79,80,81,82,83], leading to the identification of a series of 3-cyanophenyl derivatives with improved antimycobacterial properties. Most notably, some of them were also active against the homolog enzyme of the non-tuberculous opportunistic pathogen, *M. abscessus* (Figure 6H) [82,83].

Chorismic acid is also the precursor of other siderophores, produced by different bacterial pathogens through non-ribosomal peptide synthetases. A notable example is pyochelin, a siderophore produced by *P. aeruginosa* and by several members of the *Burkholderia cepacia* complex (Bcc) [84,85]. Differently from mycobacteria, the first step, namely the conversion of chorismate to salicylate is catalyzed by two different enzymes, the isochorismate synthase PchA and the isochorismate pyruvate lyase PchB. Then, PchD activates the salicylate by adenylation and transfers it to the thiol moiety of the phosphopantetheinyl group of PchE, the first enzyme of the non-ribosomal peptide synthetase complex [84]. PchD belongs to the same family of the adenylating enzyme MbtA. Very recently, its crystal structure in complex with salicyl-AMS has been solved, paving the way for the design of novel inhibitors [86].

**Figure 7:**
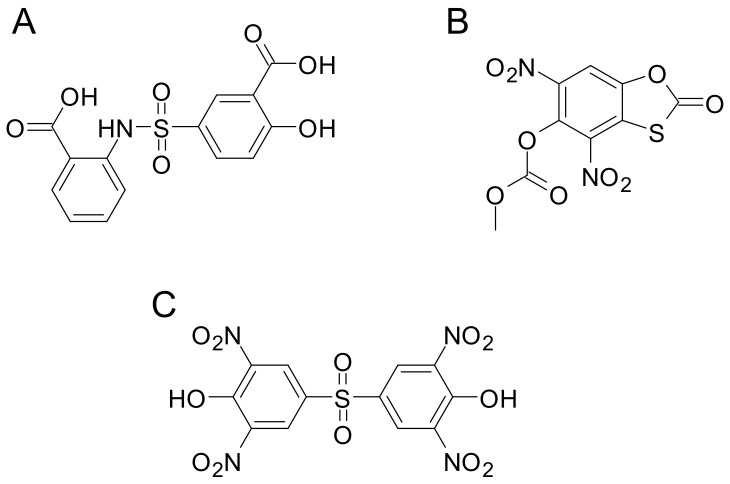
Chemical structures of compounds active against *P. aeruginosa* isochorismate pyruvate lyase PchB. Compound (**A**) was also active against *E. coli* chorismate mutase EcCM, compound (**B**) against the *Yersinia enterocolitica* salicylate synthase Irp9, and compound (**C**) against all these enzymes.

Regarding PchA, to our knowledge, no inhibitors have been reported so far. By contrast, a high-throughput screening against PchB disclosed three compounds inhibiting the isochorismate pyruvate lyase activity in the sub-micromolar range (Figure 7). However, its efficacy against *P. aeruginosa*, in iron-limiting conditions, was only in the millimolar range [86]. Interestingly, some of these compounds were also active against the salicylate synthase, Irp9 from *Yersinia enterocolitica*, and against the chorismate mutase, EcCM from *E. coli*, suggesting the potential suitability of these scaffolds for the development of specific antimicrobials [87].

**Figure 8:**
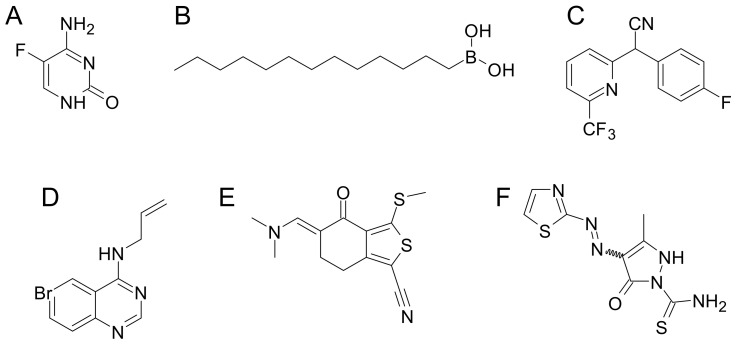
Chemical structures of the principal pyoverdine inhibitors: (**A**) the biosynthesis inhibitor 5-fluorocytosine; (**B**–**D**) *n*-alkylboronic acid, biaryl nitrile and quinazolamide PvdQ inhibitors; (**E**,**F**) pyoverdine quenchers.

Another important siderophore from the *Pseudomonas* species is pyoverdine, which consists of a dihydroquinoline-type chromophore, with a variable peptide tail. Besides being a siderophore, pyoverdine is a key virulence determinant involved in the regulation of several virulence factors important for infection, such as exotoxin A, a translational inhibitor, and the protease PrpL [88]. Interestingly, it has been demonstrated that the inhibition of pyoverdine production strongly diminishes the virulence of *P. aeruginosa* in animal models of infection [89,90]. In this context, one of the first successful examples was a drug-repurposing approach that exploited a specific biosensor for pyoverdine inhibitors to screen a library of marketed drugs. In this study, 5-fluorocytosine (Figure 8A) was shown to repress the production of pyoverdine, leading to a significant reduction of the pathogenicity [90].

The biosynthetic pathway of pyoverdine is very complex, involving at least four non-ribosomal peptide synthetases and 10 other enzymes, either cytoplasmic or periplasmic [91]. In detail, the precursor ferribactin is produced in the cytoplasm, acylated and exported to the periplasm. Here, acylated ferribactin is deacylated by the hydrolase PvdQ, and then converted into dihydropyoverdine through an oxidative cyclization, catalyzed by PvdP [91]. These two enzymes are of particular interest because they represent the two main targets of pyoverdine biosynthesis inhibitors [92,93,94,95,96]. For instance, a rational design of transition-state analogs led to the identification of potent *n*-alkylboronic acid inhibitors of PvdQ (Figure 8B), active in the nanomolar range on the enzyme, and showing good activity against *P. aeruginosa* in iron-limiting conditions [94].

Other interesting PvdQ inhibitors with different scaffolds were identified through high-throughput screening studies (Figure 8C,D), although their efficacy against the enzyme was lower than that of *n*-alkylboronic acids [95,96]. Moreover, they exhibited a decreased antimicrobial activity due to them being substrates of efflux pumps [96].

Interestingly, pyoverdine itself was found to be druggable; hence, several small molecules have been developed to directly target this siderophore. Interestingly, its chromophore core has a characteristic fluorescence at 460 nm, upon excitation at 405 nm, which is rapidly quenched when pyoverdine binds iron. This feature has thus been exploited for the high-throughput screening of potential binders, leading to the identification of molecules able to significantly reduce the virulence of *P. aeruginosa* in in vivo models, and showing synergistic effects with other antimicrobials (Figure 8E,F) [97,98,99]. In another study, to better understand how the quencher bound the siderophore, NMR spectroscopy was employed to identify the ligand-binding site [99]. Through molecular docking and molecular dynamic simulations, this investigation allowed for constructing a structural model of pyoverdine in complex with a ligand, useful for the development of more potent and specific inhibitors [98].

Most notably, it was recently reported that the inhibition of pyoverdine biosynthesis has synergistic effects with gallium nitrate, further confirming the efficacy of interfering with iron metabolism at different levels to reduce virulence and pathogenicity [99].

### 3.4. Exploiting Siderophore Uptake Systems to Deliver Antimicrobials

Common issues of antimicrobials are their low permeability across bacterial membranes and potential cytotoxicity against human cells. In this context, the use of suitable delivery vectors could significantly help in optimizing the efficacy of antibiotics [100]. Since human cells do not use siderophores, the specific bacterial uptake systems could be exploited to selectively internalize antibiotics inside the pathogens, by conjugating them to specific siderophores [100]. This strategy is naturally exploited by some microorganisms, such as *Streptomyces* and *Actinomyces*, that produce sideromycins, i.e., siderophores conjugated with an antimicrobial moiety, to compete with other species.

On this basis, different siderophore–drug conjugates (SDGs), targeting different bacteria, have been synthesized [101]. The most exploited drugs are β-lactams, particularly cephalosporins, but other classes of compounds have also been considered. For instance, several groups synthesized siderophores conjugated with fluoroquinolones [102,103,104] or macrolides [105] active against *Pseudomonas* and *Burkholderia* spp., as well as with glycopeptides [106] and lipopeptides [106,107], displaying good antimicrobial activity against *Acinetobacter baumanii*.

SDG may have either a non-cleavable or a cleavable linker between the drug and the siderophore, which can confer advantages, but also lead to some disadvantages. For example, a non-cleavable linker is resistant to the activity of β-lactamases, which, in some cases, may be unwanted; at the same time, the bulky molecules could have difficulties crossing the cell membrane. By contrast, a cleavable linker is useful to release the drug in situ, but may suffer from a premature cleavage before reaching the target site [108].

**Figure 9:**
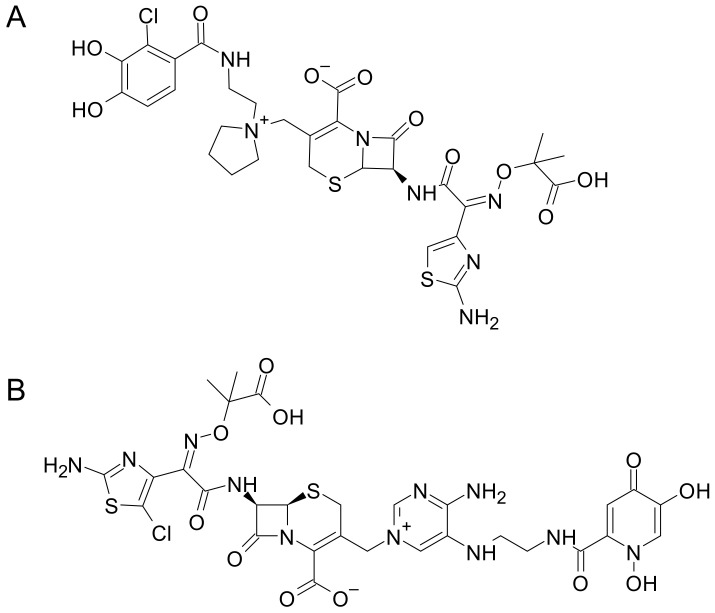
Chemical structures of the siderophore–cephalosporin conjugates, Cefiderocol (**A**) and GT-1 (**B**).

Among the siderophore-β-lactam conjugates, the most promising is the catecholate siderophore–cephalosporin Cefiderocol (Figure 9A), which showed a significant antibacterial activity against different Gram-negative bacteria (*P. aeruginosa*, *Acinetobacter baumannii*, *Klebsiella pneumoniae*, *E. coli*) in vitro, [109] multi- and extensively drug-resistant *E. coli* strains [110], carbapenem-resistant *Enterobacterales* [111], and *Burkholderia pseudomallei* clinical isolates [112]. Most notably, Cefiderocol exhibited a significant in vivo efficacy against MDR *P. aeruginosa* in a mouse infection model [109], and displayed good safety and pharmacokinetic profiles [113]. Cefiderocol has been recently authorized by the FDA and is currently undergoing phase 3 clinical trials for the treatment of severe infections (pneumonia, bloodstream infections, complicated urinary tract infections, and sepsis), caused by carbapenem-resistant Gram-negative pathogens [114]. Moreover, a phase 3 trial (APEKS-NP) demonstrated that Cefiderocol was non-inferior to high-dose meropenem for the outcome of day-14 all-cause of mortality in patients with Gram-negative pneumonia, suggesting its potential efficacy for the treatment of nosocomial infections [115]. However, the (CREDIBLE-CR) phase 3 trial evidenced a higher number of deaths in patients with *Acinetobacter* spp. infections, limiting the treatment options of Cefiderocol [116]. Different studies are ongoing to explore the efficacy of possible combinations with other antimicrobials [117,118].

Other cephalosporin–siderophore conjugates are currently under investigation. Among them, GT-1, a novel siderophore-dihydroxypyridone conjugated to a modified aminothiazolylglycyl cephalosporin (Figure 9B), showed a strong potency towards different Gram-negative pathogens, including MDR *E. coli*, *K. pneumoniae*, and *Acinetobacter* spp. isolate strains [119]. Furthermore, GT-1 proved to be resistant to the hydrolytic activity of different bacterial β-lactamases and carbapenemases. Moreover, the combination of CT1 with GT-055, a β-lactamase inhibitor, enhanced the efficacy of the compound against the tested strains, including those that were resistant to GT-1 [119].

Artificial siderophores have also been implemented to exploit iron uptake systems as delivery tools for drugs [109,120,121]. The 1,3,5-N,N′,N″-Tris-(2,3-dihydroxybenzoyl)-triaminomethylbenzene (MECAM) siderophore, for example, has been conjugated through cleavable and non-cleavable linkers to different molecules (ampicillin, daptomycin, amoxicillin), potentiating their effect on different bacteria, such as *E. coli*, *S. aureus*, *A. baumanii*, and *E. faecium* [122], with the best compound showing MIC values in the nanomolar range against the considered strains. Interestingly, a study on *E. coli* showed that MECAM may exploit three different catechol receptors to reach the periplasm, namely FepA, CirA, and Fiu, and that only a triple mutant of the three proteins may confer resistance to the conjugate. This observation further highlighted the suitability of artificial siderophores to develop a Trojan Horses strategy to fight AMR [122].

Siderophore-conjugated compounds have also proven to be quite promising in drug-repositioning efforts [123]. Good examples are siderophore–methotrexate conjugates [124]. Methotrexate is an anticancer drug that inhibits human dihydrofolate reductase (DHFR), an essential enzyme in nucleotide biosynthesis. This compound was also found to be active against several bacterial DHFR, sometimes with an even higher affinity compared to the human isoform. However, its low permeability across bacterial membranes, as well as its cytotoxicity against human cells, prevented its use as an antimicrobial agent. To overcome this issue, methotrexate was conjugated with an analog of the hydroxamate siderophore, ferrichrome, using different non-cleavable linkers. The best conjugate exhibited considerable activity against *Streptococcus pneumoniae* and *Y. enterocolitica*, with an MIC in the nanomolar range. Moreover, it did not show toxicity against mammalian cells. Interestingly, the compound was not active against other Gram-negative and Gram-positive bacteria, such as *Acinetobacter baumannii*, *Staphylococcus epidermidis* or *Salmonella enterica*, suggesting the possibility of developing narrow-spectrum antibiotics targeting a specific bacterium [124].

## 4. Conclusions and Future Outlook

Virulence factors play important roles in the pathogenic process of microorganisms, mediating bacterial adhesion and colonization, host immune suppression, and immune escape. Hence, anti-virulence treatments, aimed at reducing the pathogenicity, while sparing the bacterium for its eventual elimination by the immune system or other therapies, may have significant advantages over traditional approaches. Most notably, the reduced selective pressure on bacteria would limit the emergence of AMR. Therefore, considering the ever-increasing spread of drug-resistant strains, anti-virulence therapy is expected to become a crucial tool to fight infections and, as such, worthy of extensive research. In this review, we focused on new potential pharmacological targets involved in the pathways of iron acquisition and metabolism. For each class, we reported the most interesting molecules that have been developed to interfere with these mechanisms, highlighting their biological activity and pharmacokinetic/toxicological profile.

Among the different strategies, the ability of gallium to act as an iron competitor has emerged as a particularly promising option. Ga(III)-based treatments have shown effective antimicrobic properties against different Gram-negative and Gram-positive bacteria, including ESKAPE species. Most notably, they have also exhibited antibacterial action against resistant strains. However, some limitations to their use still exist, including the low bioavailability and uncontrolled release. Hence, current research is focused on the optimization of the delivery. In this context, nanomaterials have yielded very promising results so far.

Another very effective approach is to hinder iron acquisition by interfering with the production of siderophores. Considering that these molecules are absent in humans, they are ideal targets for the design of safe antimicrobial drugs. In *M. tuberculosis*, this strategy has produced interesting results, with several effective compounds being developed as inhibitors of MbtA and MbtI. In detail, furan-based inhibitors of MbtI have proven to be particularly attractive for their activity in both *M. tuberculosis* and *M. abscessus*, an emerging NTM. In *Pseudomonas* spp., the investigation of the siderophore pyoverdine has allowed the discovery of various inhibitors acting by different mechanisms, able to significantly reduce the bacterial virulence. Moreover, siderophores may also be exploited to enhance the internalization of antibiotics inside bacteria by the development of conjugates. Of note, the catecholate siderophore–cephalosporin, Cefiderocol, has recently obtained the FDA approval and is currently undergoing phase 3 clinical trials for the treatment of severe infections caused by carbapenem-resistant Gram-negative pathogens. Artificial siderophores have also been implemented to design more effective conjugates, exploiting this Trojan Horse strategy.

Overall, this review demonstrates that iron homeostasis is a very promising source of innovative molecular targets for the development of anti-virulence compounds against a variety of bacterial species. Although there are still many gaps in our knowledge of these systems, the fundamental understanding of iron acquisition and metabolism in bacteria should enable new, well-reasoned approaches to develop better therapeutic strategies to fight AMR.

## Data Availability

No new data were created or analyzed in this study. Data sharing is not applicable to this article.
